# Do wild-caught urban house sparrows show desensitized stress responses to a novel stressor?

**DOI:** 10.1242/bio.031849

**Published:** 2018-04-09

**Authors:** Noraine Salleh Hudin, Aimeric Teyssier, Johan Aerts, Graham D. Fairhurst, Diederik Strubbe, Joël White, Liesbeth De Neve, Luc Lens

**Affiliations:** 1 Terrestrial Ecology Unit, Department of Biology, Ghent University, K.L. Ledeganckstraat 35, B-9000 Ghent, Belgium; 2 Department of Biological Sciences, Faculty of Science & Mathematics, Universiti Pendidikan Sultan Idris, 35900 Tanjong Malim, Perak, Malaysia; 3 Stress Physiology Research Group, Faculty of Pharmaceutical Sciences, Ghent University, Wetenschapspark 1, 8400 Ostend, Belgium; 4 Stress Physiology Research Group, Animal Sciences Unit, Flanders Research Institute for Agriculture, Fisheries and Food, Wetenschapspark 1, 8400 Ostend, Belgium; 5 Department of Biology, University of Saskatchewan, 112 Science Place, Saskatoon, Saskatchewan S7N 5E2 Canada; 6 Laboratoire Evolution & Diversité Biologique, UMR 5174 CNRS-Université Paul Sabatier-IRD-ENSFEA, 118 route de Narbonne, F-31062 Toulouse, France

**Keywords:** Feather corticosterone, Passerines, Aviary, Urban exploiter, Moult

## Abstract

While urbanization exposes individuals to novel challenges, urban areas may also constitute stable environments in which seasonal fluctuations are buffered. Baseline and stress-induced plasma corticosterone (cort) levels are often found to be similar in urban and rural populations. Here we aimed to disentangle two possible mechanisms underlying such pattern: (i) urban environments are no more stressful or urban birds have a better ability to habituate to stressors; or (ii) urban birds developed desensitized stress responses. We exposed wild-caught urban and rural house sparrows (*Passer domesticus*) to combined captivity and diet treatments (urban versus rural diet) and measured corticosterone levels both in natural tail feathers and in regrown homologous ones (cort_f_). Urban and rural house sparrows showed similar cort_f_ levels in the wild and in response to novel stressors caused by the experiment, supporting the growing notion that urban environments are no more stressful during the non-breeding season than are rural ones. Still, juveniles and males originating from urban populations showed the highest cort_f_ levels in regrown feathers. We did not find evidence that cort_f_ was consistent within individuals across moults. Our study stresses the need for incorporating both intrinsic and environmental factors for the interpretation of variation in cort_f_ between populations.

## Abstract (precis)

**Summary:** Corticosterone in natural and regrown feathers was similar between urban and rural birds. Sex and age related to corticosterone in regrown feathers. Feather corticosterone was not consistent across different moults.

## INTRODUCTION

As urban environments continue to expand and encroach into natural ones, understanding how organisms cope with anthropogenic disturbance becomes increasingly important in ecological and evolutionary studies, and when aiming to draft sustainable conservation strategies (e.g. Marzluff, 2001; Shochat et al., 2006; Ellis et al., 2012; Magle et al., 2012; Alberti, 2015). Urbanization exposes individuals to abiotic and biotic challenges, such as high human activity, noise and light pollution, toxins, endocrine disrupting chemicals, and non-native predators or diseases (e.g. Bichet et al., 2013; Bradley and Altizer, 2007; Grimm et al., 2008; Lowry et al., 2013). An expanding body of literature on urban ecology has identified general characteristics that enable some species to colonize and thrive in cities nonetheless (Bonier et al., 2007a; Concepción et al., 2015; Davey et al., 2012; Evans et al., 2011; Kark et al., 2007; Williams et al., 2015). For certain species, urban habitats may further constitute stable environments that buffer seasonal fluctuations in resource availability and other sources of stochasticity that characterize most natural environments (Anderies et al., 2007; Fokidis et al., 2008).

Birds, like other vertebrates, produce a coordinated endocrine response when faced with acute or chronic stressors (Sapolsky et al., 2000). An immediate adrenaline response prepares the organism for the ‘fight or flight’ reaction, followed by a release of glucocorticoids such as corticosterone (cort) via activation of the hypothalamic-pituitary-adrenal (HPA) axis (Bonier, 2012; McEwen and Wingfield, 2010; Sapolsky et al., 2000). Plasma glucocorticoids are generally considered to mediate a redistribution of energy (i.e. glucose) with the ultimate goal of restoring homeostasis (Breuner et al., 2008; Kitaysky et al., 2001; Romero, 2004). Yet individuals may habituate to repeated stressors by learning that these are harmless and can be ignored (Romero, 2004). If such acclimation occurs, the HPA axis may not be activated when exposed to these particular stressors, but the individual maintains the full ability to respond to novel ones (Cyr and Romero, 2009). Alternatively, repeated challenges may cause alterations in HPA activity and attenuate the release of cort in response to stressors without the perception that a challenge is no longer threatening [desensitization sensu (Cyr and Romero, 2009; Rich and Romero, 2005)]. In such cases, environmental stressors can affect the organism-wide stress physiology which may incur costs if it comprises an individual's ability to respond to ecologically-relevant stressors (Cyr and Romero, 2009). If stress continues to increase, the physiological system may eventually break down and cort release may decrease as the stress response can no longer be maintained (Cyr and Romero, 2009).

In house sparrows (*Passer domesticus*), a common urban-dwelling passerine, several studies reported comparable baseline cort levels in blood plasma of urban and rural populations, at least during the non-breeding season (Bókony et al., 2012; Chavéz-Zichinelli et al., 2010; Fokidis et al., 2009; Meillère et al., 2015). Such a pattern could indicate that urban environments are not experienced as any more stressful than rural ones or urban sparrows habituate to known local stressors and thereby maintain a similar stress physiology to rural ones (Bonier, 2012; Cyr and Romero, 2009), or (ii) that urban sparrows exhibit desensitization and have developed a HPA axis physiology with dampened responses to novel stimuli (Atwell et al., 2012; Cyr and Romero, 2009). To experimentally test whether urban sparrows maintained a similar stress physiology compared to rural sparrows or developed desensitization, we exposed wild-caught urban and rural house sparrows to a combined (i.e. captivity and diet treatment) challenge experiment in a full-factorial design, and measured cort levels in original (i.e. grown under natural conditions prior to capture) and regrown (i.e. grown under experimental conditions after induced moult) tail feathers. Earlier studies found evidence for increased baseline and stress-induced plasma glucocorticoid concentrations due to captivity, even when sampled after several weeks (Cyr and Romero, 2008; Marra et al., 1995; Fischer et al., 2018). Diet treatments mimicked either a rural or an urban diet, and diet analysis confirmed that both regimes significantly differed in key nutrients [full details in Salleh-Hudin et al., (2016)]. Captive individuals can thereby be predicted to experience the highest chronic stress when supplied with a diet that deviates most strongly from their natural one. Feather cort (cort_f_) provides a long-term integrated measure of baseline (due to circadian rhythmicity), acute and chronic levels in response to allostatic demands during feather synthesis (Johns et al., 2018; reviewed in Romero and Fairhurst, 2016). In our study set-up, cort_f_ values measured in original feathers reflect responses to pre-capture environmental conditions, while those in regrown ones signal an individual's ability to cope with novel, experimental stressors (Cook, 2012). In addition to cort_f_, two other proxies of environmental and nutritional stress were measured on the same individuals, i.e. (i) fluctuating asymmetry (FA) in tail feather length and (ii) tail feather growth bar widths (GBW). FA refers to small random deviations from perfect symmetry and reflects the level of exposure to developmental stress or of an individual's intrinsic ability to cope with it [lower FA signaling lower developmental stress; (Lens et al., 2002)], while GBW uses daily feather growth rates to assess an individual's nutritional stress during the period of feather growth [larger GBW signaling better nutritional conditions; (Grubb and Cimprich, 1990; Vangestel and Lens, 2011)].

We predicted cort_f_, FA and GBW in regrown feathers to be similar between urban and rural sparrows if urban birds did not alter their stress physiology (Table 1). If so, we also expected lower cort_f_ and FA, and larger GBW, in sparrows fed on their own (mimicked) diet than those fed on the alternative one, irrespective of their urban or rural origin (Table 1). Alternatively, if urban sparrows developed desensitization (and hence attenuated stress responses), cort_f_ and FA in regrown feathers of urban sparrows was expected to be lower, and GBW to be larger, compared to regrown feathers of rural ones, while a diet change was predicted to affect rural birds more strongly than urban ones (Table 1).

**Table 1.**
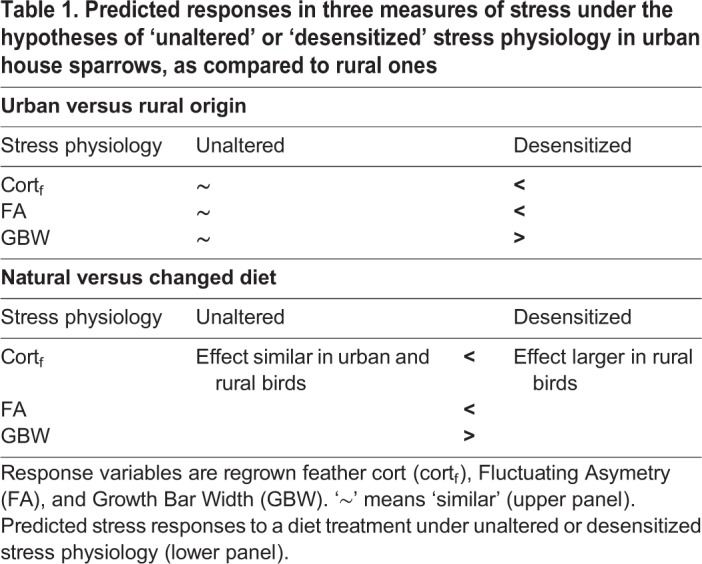
**Predicted responses in three measures of stress under the hypotheses of ‘unaltered’ or ‘desensitized’ stress physiology in urban house sparrows, as compared to rural ones**

## RESULTS

### Variation in original feathers (pre-experimental)

**Fig. 1.**
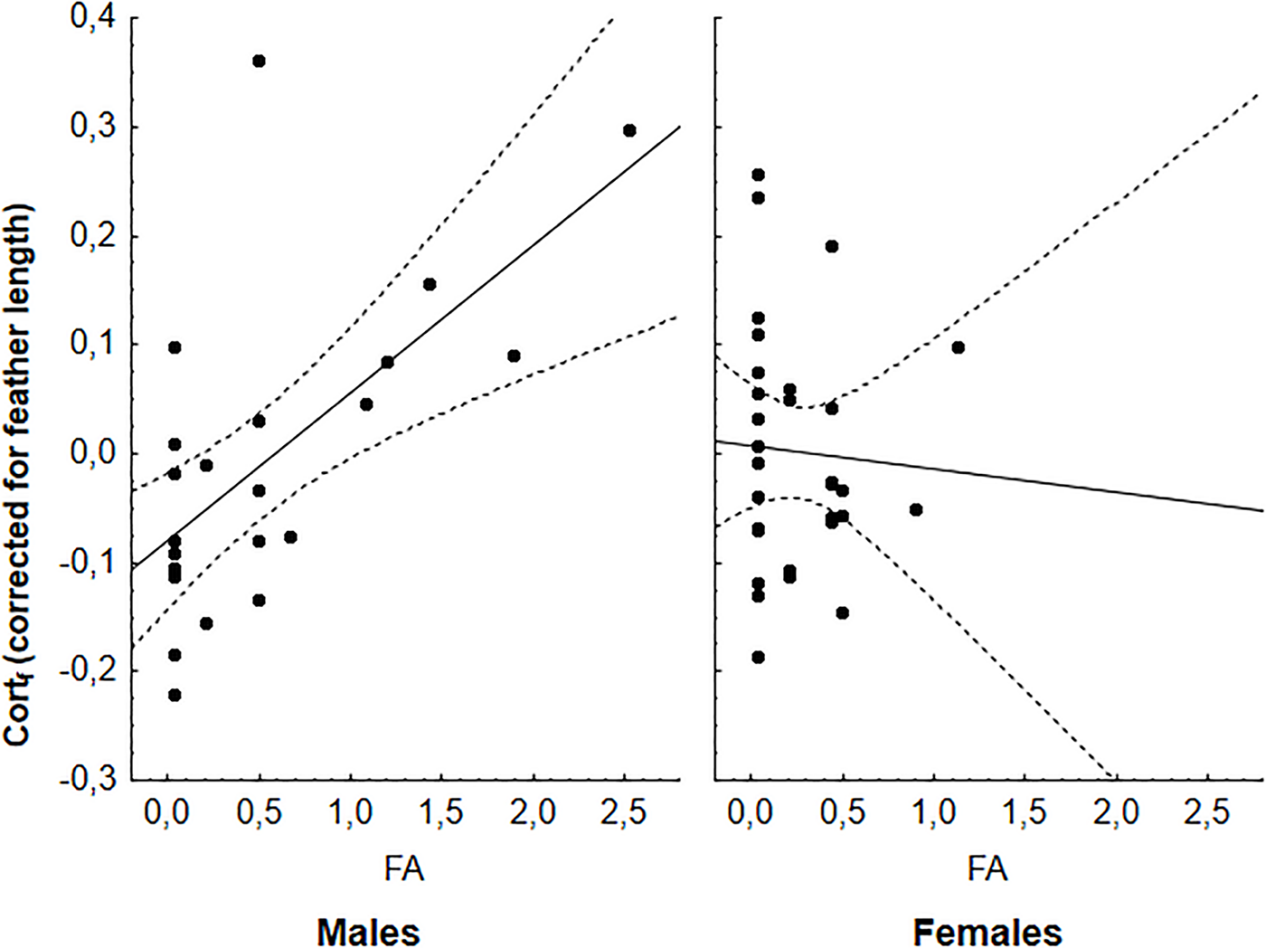
**cort_f_** (**corrected for feather length) in relation to FA in males and females.** Full model results can be found in **Table S2**.

**Fig. 2.**
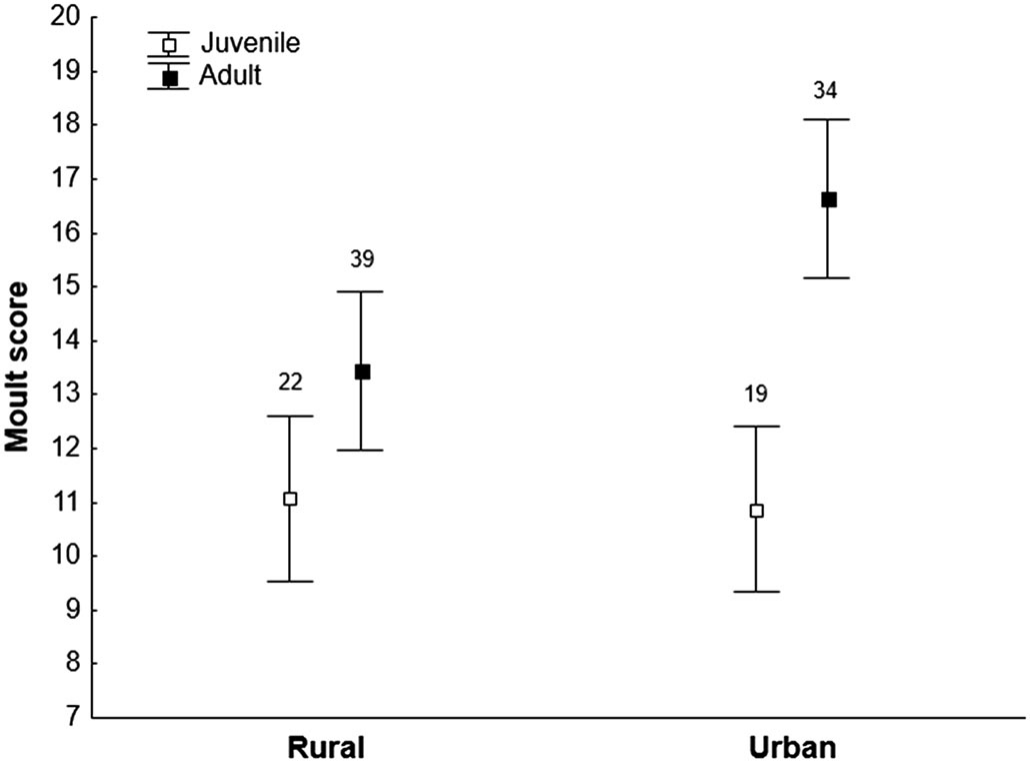
**Average cumulative moult scores (±s.e.m.) in juveniles and adults from urban and rural populations.** Moult score ranges from 6–18, and higher scores indicate advanced moult (see the Materials and Methods). Sample sizes in each group are also shown.

Cort_f_ of original feathers did not significantly differ between urban and rural populations, nor between sexes or ages (all *P*>0.11, Table 2). FA was positively related to cort_f_ in males but not in females (sex×FA: *F*_1,48_=3.63, *P*=0.063, Table S2; Fig. 1), while GBW and all interactions with urbanization, sex or age did not significantly explain variation in cort_f_ (all *P*>0.25, Table S3). Cumulative moult scores of original feathers significantly differed between urban and rural populations in interaction with age (urbanization×age, *F*_1,108_=8.51, *P*=0.0043, Fig. 2), but did not differ between sexes (*F*_1,105_=0.06, *P*=0.81). Adult sparrows in urban populations initiated their moult earlier than those in rural ones, while most juveniles were not yet moulting at the time of sampling (Fig. 2). As a consequence, moult state of original removed feathers was unevenly distributed among populations and ages (see Fig. S1).

**Table 2.**
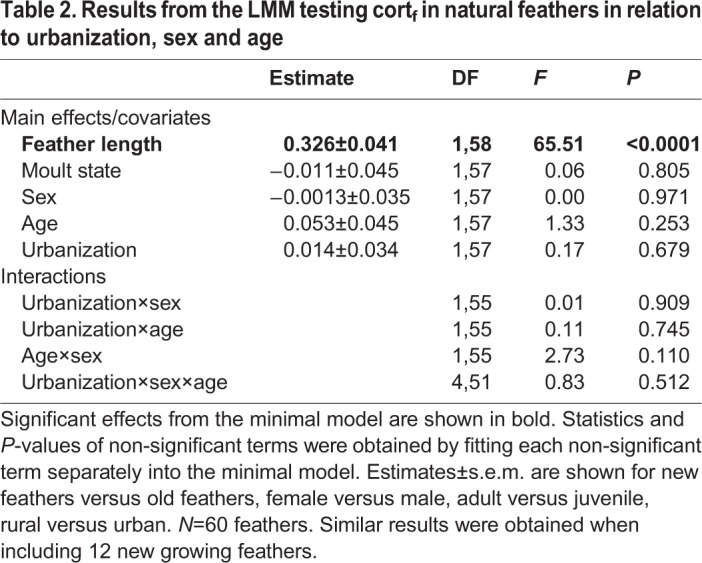
**Results from the LMM testing cort_f_ in natural feathers in relation to urbanization, sex and age**

### Variation in regrown feathers (post-experimental)

**Fig. 3.**
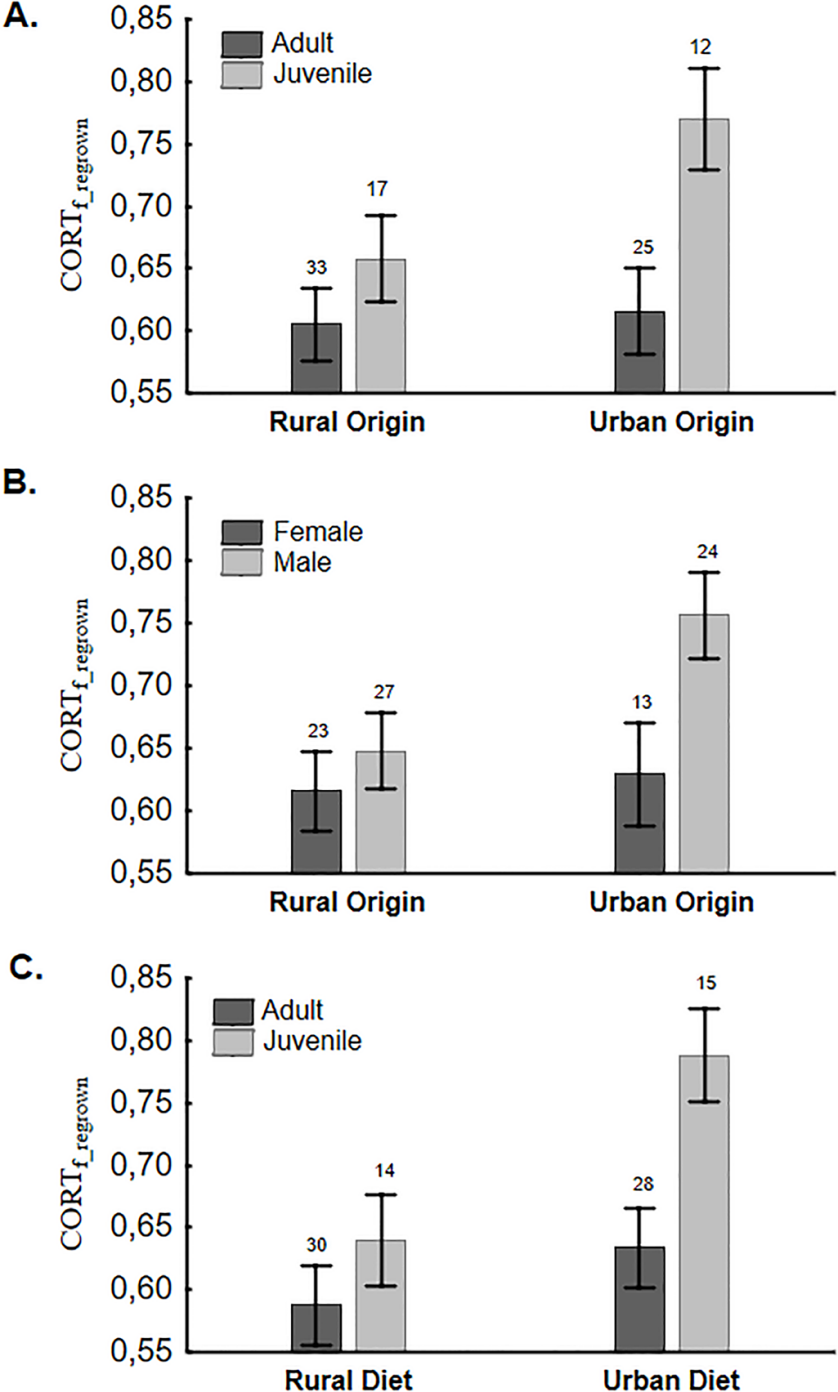
**Variation in cort of regrown feathers (cort_f_regrown,_ mean±s.e.m.) in relation to significant interactions by (A) urbanization and age, (B) urbanization and sex, and (C) diet and age.** Full model results can be found in Table 2. Sample sizes in each group are shown.

Variation in cort_f_ of regrown feathers interacted with the level of urbanization in the population of origin, diet, sex and age (i.e. significant urbanization×age, urbanization×sex and diet×age interactions) (Table 3, Fig. 3). Overall, juvenile sparrows showed higher values than adults, in particular those originating from urban populations and exposed to an urban diet (Fig. 3A,C, Table 3). Likewise, urban males showed higher values than females (Fig. 3B, Table 3). Within individuals, cort_f_ values of original and regrown feathers did not differ (repeated measures, *F*_1,54_=1.016, *P*=0.318), and were not correlated (R^2^=0.0004, *P*=0.88). Cort_f_ of regrown feathers was not correlated with FA or GBW, and there were no interactions with sex, age, urbanization or diet (FA model, all *P*>0.31; GBW model, all *P*>0.13).

**Table 3.**
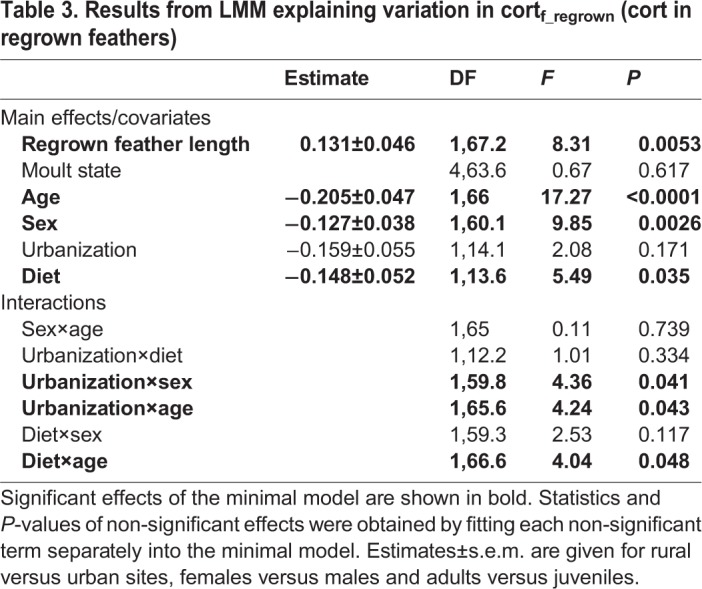
**Results from LMM explaining variation in cort_f_regrown_ (cort in regrown feathers)**

**Fig. 4.**
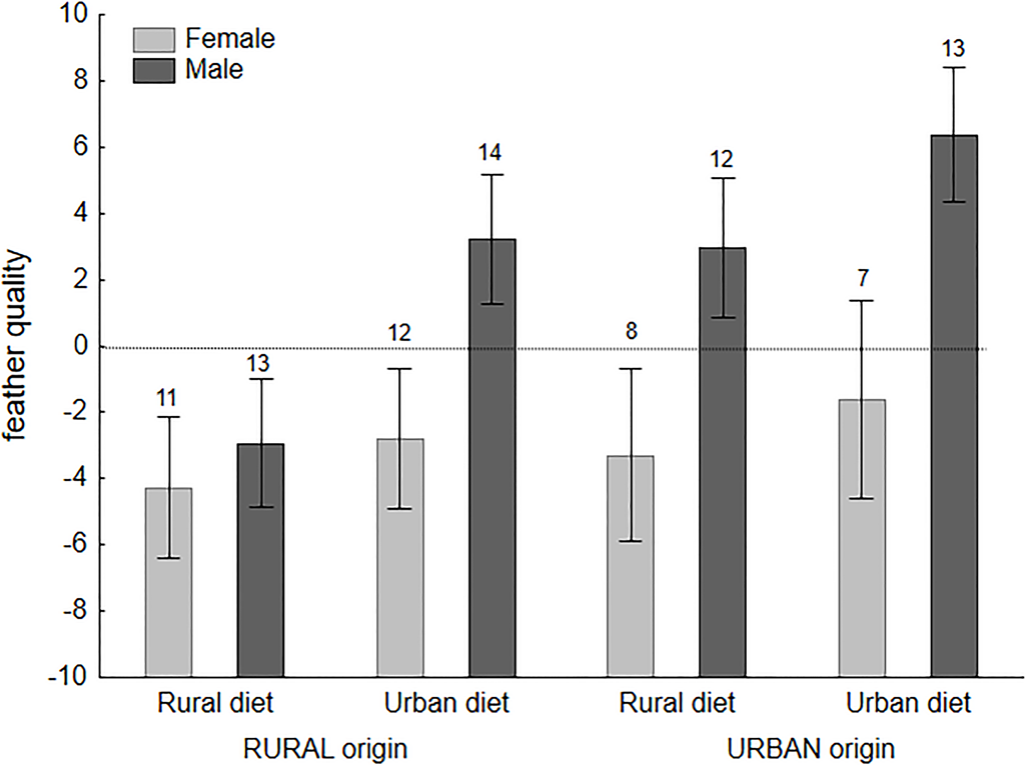
**Average regrown feather quality (±s.e.m.) in males and females from rural and urban sites in relation diet treatment.** Sample sizes are also shown.

**Fig. 5.**
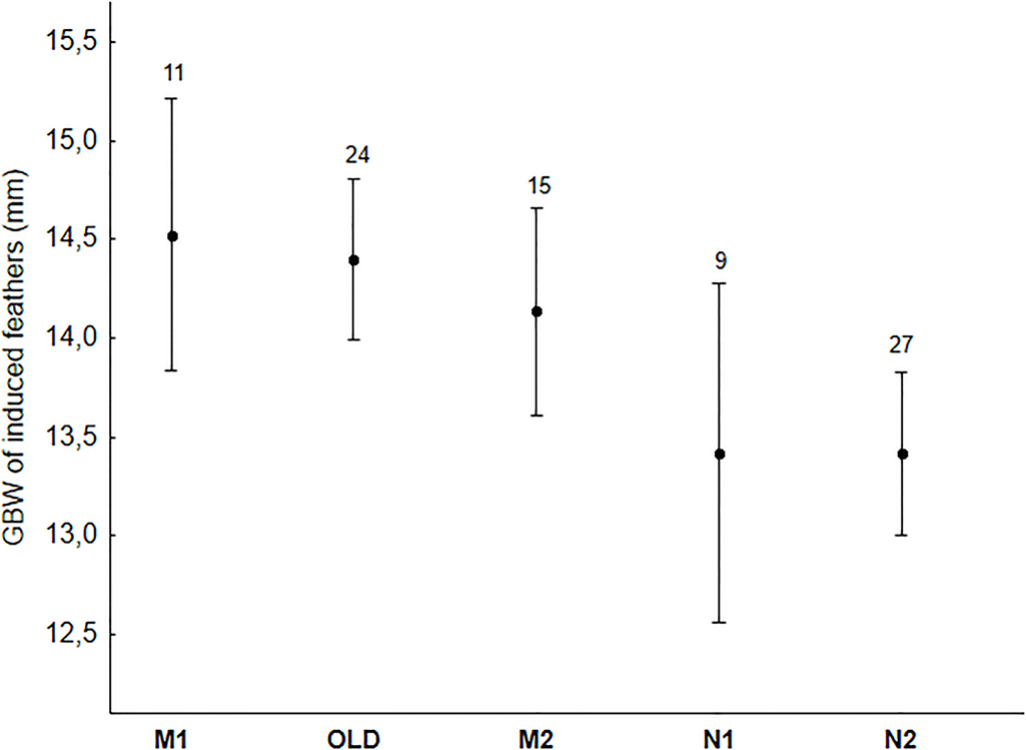
**Average (±95% C.I.) GBW of regrown feathers according to moult state of the removed feather.** Values are corrected for variation explained by feather length and growth time (see text). Moult states are presented in order of increasing costs that already had been invested in moult: M_1_, Old feather is dropped, but moulting feather is too small to be plucked; O, Old feather; M_2_, Moulting feather, <50% grown and large enough to be plucked; N_1_, New feather, >50% but not full grown; N_2_, New feather full grown.

Variation in quality of regrown feathers was significantly explained by the diet treatment (*F*_1,14.9_=4.91, *P*=0.042) and sex (*F*_1,70.1_=12.64, *P*=0.0007) and marginally explained by the level of urbanization in the population of origin (*F*_1,15.1_=3.93, *P*=0.066, see Table S4 for full model results), but not explained by moult state of the original feather (*F*_4,74.5_=2.00, *P*=0.103). Male sparrows grew feathers of higher quality than females, and individuals from an urban origin that were kept on an urban diet, grew higher quality feathers than those from all other origin×diet combinations (Fig. 4). Variation in GBW of regrown feathers was marginally explained by the moult state of their natural homologues (*F*_4,68.2_=2.43, *P*=0.055, Fig. 5), after controlling for length (*F*_1,70.3_=10.08, *P*=0.0022, estimate 1.24±0.39) and growth time (*F*_1,67.4_=21.35, *P*<0.001, estimate −0.76±0.16). The largest GBW and steepest growth rates were measured in naturally regrown feathers (category M_1_, see the Materials and Methods) and in old feathers. In contrast, regrown feathers induced by plucking naturally-moulted new ones at the onset of the experiment, showed the slowest growth (Fig. 5). FA of regrown feathers was positively correlated with its growth time (LMM *F*_1,74_=6.32, *P*=0.014, estimate 0.28±0.11) but was not affected by moult state (*F*_4,65.8_=1.41, *P*=0.24). When comparing natural and regrown feathers of the same individuals, the latter were shorter, lighter and had lower GBW values (repeated measures, all *P*<0.0001).

## DISCUSSION

No descriptive or experimental studies to date have provided conclusive evidence that urban-dwelling passerines such as house sparrows exhibit different HPA axis functioning compared to their rural counterparts (Bókony et al., 2012; Chavéz-Zichinelli et al., 2010; Fokidis et al., 2009; Meillère et al., 2015), while evidence is growing that HPA axis functioning is plastic and variable among ages and sexes (Aharon-Rotman et al., 2017; Dantzer et al., 2014). Building on these emerging patterns, we predicted that a novel stressor, simulated by a combined captivity and diet treatment, would affect indicators of stress of urban and rural sparrows in similar ways. Results of our study do confirm that urban and rural individuals had comparable levels of corticosterone in feathers moulted naturally, suggesting that allostatic load during moult did not differ in relation to urbanization. Despite this general pattern, subtle differences emerged in the two groups following captivity and diet treatment. Individuals from urban origin tended to have slightly higher cort_f_ levels in their regrown feathers compared to rural sparrows, although not significantly so and confounded by age and sex. This non-significant trend was in the opposite direction of what would be expected if urban birds habituated sooner to captivity or developed a change in HPA axis reactivity [e.g. desensitization with reduced capacity to react to a novel stressor; (Cyr and Romero, 2009)]. Hence, our study of longer-term (i.e. during feather growth) HPA functioning supports growing notion, based on short-term baseline plasma cort levels reported previously, that for house sparrows cities are not necessarily any more challenging during the non-breeding season than are rural areas (Bókony et al., 2012; Chavéz-Zichinelli et al., 2010; Fokidis et al., 2009; Meillère et al., 2015).

Cities may provide urban-dwelling species with favorable foraging opportunities, at least for adults, which appears to contradict the common finding that urban sparrows are often leaner than rural ones (Bókony et al., 2012; Meillère et al., 2015; Salleh Hudin et al., 2016). Yet, experimental studies suggest that the lower body condition of urban sparrows likely reflects a plastic response to more predictable food supplies, trading-off with predation risk (Dulisz et al., 2016; Salleh Hudin et al., 2016). In line with this, no differences in GBW or feather quality were found between urban and rural house sparrows [(Meillère et al., 2017; Salleh Hudin et al., 2016), this study], two traits assumed to be affected by diet quality (Pap et al., 2008). Studies of behavioral coping styles also predicted urban sparrows to be bolder than rural ones (Atwell et al., 2012; Lendvai et al., 2011), although a recent experimental study could not confirm this (Vincze et al., 2016). Irrespective of the underlying mechanism(s) involved, urban environments appear to fulfil the energetic requirements of house sparrows quite well, which may explain why urban exploiters do not change their HPA axis functioning in response to urbanization, unlike some urban adaptor species (Dantzer et al., 2014). Urban house sparrows in our study did initiate moult earlier compared to rural populations, which is in agreement with results from a recent study on the Carolina chickadees [*Poecile carolinensis*; (Hope et al., 2016)]. An earlier onset of moult in urban environments is likely a carry-over effect from earlier breeding (Chamberlain et al., 2009), given that the onset of moult is often correlated with the end of reproduction (Siikamaki et al., 1994; Vega Rivera et al., 1998).

The few studies that implicitly modelled intrinsic factors in stress physiology [reviewed in Dantzer et al., (2014)] revealed age- and sex-related variation in phenotypic plasticity in glucocorticoid levels, highlighting the value of taking these factors into account when comparing patterns across studies (Aharon-Rotman et al., 2017). Significant differences between sexes and ages in cort_f_ levels of experimentally regrown feathers [but not in naturally moulted ones; see also Koren et al., (2012)] found in our study confirm the latter: when challenged by captivity and diet treatments, juveniles apparently secreted more cort during feather growth than adults, and males secreted more than females (Fig. 3). Adding to the level of complexity, the strength of these intrinsic effects also varied with the level of urbanization in the population of origin, as juveniles and males originating from urban populations had the highest cort concentrations in their regrown feathers (Fig. 3). It is possible that stronger cort responses are adaptive for less experienced individuals [such as juveniles; (Boves et al., 2016)], as a higher HPA activity may increase their reactivity, and hence, their success in escaping from life-threatening events. In support of this, juvenile male house sparrows were the most difficult group to trap in an aviary experiment on sex- and age-related differences in escape response, and escape ability related to stress response, which was overall higher in juvenile birds (De Neve et al., 2010). Similarly, Rivers et al., (2012) found that higher baseline cort levels in fledgling Swainson's thrushes (*Catharus ustulatus*) was related to increased post-fledging survival rates, which was suggested to be mediated by higher locomotor activity allowing better foraging and effective escape from predators. Also, Boves et al. (2016) suggested that the higher cort_f_ levels of juvenile cerulean warblers (*Setophaga cerulean*) measured during post-breeding, and again during the subsequent non-breeding period, reflected their inexperience and increased foraging compared to more experienced adult males. Why, in our study, juveniles originating from urban populations showed higher levels of cort_f_ than those from rural areas, however, remains puzzling. A possible, yet speculative, explanation is that the strength and frequency of activation of the stress response of the former was mediated by a higher perceived predation risk due to the prominent presence of cats and corvids and/or the more frequent disturbance (traffic, people walking by, etc.) in cities.

With respect to gender, most studies so far reported higher effect sizes of glucocorticoid levels in males [Dantzer et al., (2014), but see Aharon-Rotman et al., (2017) for an opposite trend]. In our study we also observed that males, on average, responded more strongly to captive stress than females, in particular in individuals originating from urban populations (Fig. 3). While to the best of our knowledge, no consistent explanation exists for such sex-specific differences in responsiveness during the non-reproductive state (Dantzer et al., 2014), the complex mechanisms underlying cort deposition into feathers (Romero and Fairhurst, 2016) may offer a possible explanation. Cort_f_ is passively deposited during feather formation (Bortolotti, 2010; Jenni-Eiermann et al., 2015; Romero and Fairhurst, 2016), and concentrations are therefore likely determined by an interaction of plasma cort levels with the growth of the developing feather (Fairhurst et al., 2013; Patterson et al., 2015). High plasma cort and low-quality diet can both negatively affect feather growth and structure (DesRochers et al., 2009; Lattin et al., 2011; Pap et al., 2008; Strochlic and Romero, 2008), and hence, affect cort deposition. Furthermore, as regrown feathers do not emerge through natural moult, different physiological mechanisms or energy availability might be at play that could further affect feather development and cort deposition. The rate of induced feather regrowth (measured through GBW) was indeed related to moult state (i.e. costs already invested in natural moult). As expected, individuals that had invested less in moult had the fastest growing induced feathers. However neither moult state nor growth time significantly affected regrown feather quality. Instead, regrown feathers were of lower quality in females than in males, which is consistent with a sexual difference in tail feather quality of natural moulted feathers (Meillère et al., 2017). In line with cort_f_ results, males originating from urban populations and those on an urban diet also regrew feathers of the highest quality (Fig. 4). Hence, even after correcting for feather length or quality, differences in physiology and/or feather properties between sexes may still affect cort deposition in regrown feathers, and hence, partially explain intersexual variation in cort_f_. Another recent experimental study on house sparrows also found that sex was a significant factor affecting cort_f_ in regrown feathers. In addition, cort_f_ of regrown feathers was related to cort_f_ in natural feathers (pre-treatment), irrespective of experimental treatment (i.e. cort implants) (Aharon-Rotman et al., 2017). However, we could not confirm this individual consistency in cort_f_ across different moults as cort in natural moulted feathers (pre-treatment) was not correlated with cort in regrown feathers, and this relationship was not affected by sex, age, urbanization of moult state of the plucked feather. Experimental induction of moult in mallard (*Anas platyrhynchos*) ducklings also revealed that cort_f_ levels in regrown feathers only reflected levels in the original feathers in birds that experienced allostatic load conditions (Johns et al., 2017).

In conclusion, results of this study support the hypothesis that the overall physiological response of urban exploiters may not differ substantially across urbanization gradients, despite the fact that urban and rural environments likely present different suites of stressors. They also underscore the importance of incorporating both intrinsic and environmental factors when aiming to apply cort_f_ as a biomarker in ecological or conservation studies.

## MATERIALS AND METHODS

### Study area and sampling procedures

House sparrows were caught with mist-nets between September 18th and October 5th 2014 in three urban (Toulouse 43°36′ N, 01°26′ E; Tarbes 43°13′ N, 00°04′ E; and Pau 43°18′ N, 00°22′ E) and three rural plots (Caraman 43°31′ N, 01°44 E; Montégut-Savès 43°25′ N, 00°58′ E; and Cologne 43°42′ N, 00°55′ E) in Southern France. With ArcMap 10.2.2, urbanization levels at each trapping site were calculated as the percentage of built-up area within a 100 m radius, corresponding to the average home range size of house sparrows during the non-breeding season (Vangestel et al., 2010). MIPY Geo maps (MIPYGeo Grand Public) were available for all sites except Pau. For the latter location, we used a CORINE Land Cover map as urbanization levels calculated from both sets of maps were highly positively correlated (Pearson's r=0.96, *P*<0.001). Urbanization levels reached 100% in the three urban plots and were consistently low in the three rural ones (Caraman 3.77%; Montégut-Savès 5.03%; Cologne 3.95%). The nearest distance between two neighboring sampling plots was 25 km, which was eight times larger than the average movement range of house sparrows [<3.2 km, (Anderson, 2006) and references therein]. Although house sparrows have occasionally been observed dispersing as far as 48 km (Tufto et al., 2005), such large-distance dispersal events are very rare [<2% individuals, (Altwegg et al., 2000)] and sampling plots were therefore considered statistically independent.

A total of 53 sparrows were caught in the three urban plots and 61 sparrows in the three rural ones. Upon capture, each individual was ringed, aged (juvenile versus adult) and sexed, and the moult stage of six tail feathers (left body side) was scored as one (old feather, based on color and wear), two (active moult; <50% of the full-grown feather length), or three (new feather; >50% of the full-grown feather length). In some rare cases (1.3% of feathers) ‘no feather’ was observed, which could be due to loss during capture or when a moulting feather was not yet visible; these cases were assigned a score of two (i.e. the average score). Cumulative moult scores of all six rectrices were calculated for each bird. Next, the fifth (i.e. second outermost) right and left rectrix of each bird was plucked and individually stored at room temperature. For each of the removed feathers, a ‘moult state’ category was assigned: (i) ‘Old feather’ (O); (ii) ‘Moulting feather’, which were either too small to pluck (M_1_–moulting but not plucked) or too small for cort_f_ analysis (M_2_–moulting plucked); (iii) ‘New feather’ (>50% of the full-grown feather length), which were either nearly fully grown (N_1_) or fully grown (N_2_). Feathers from which we could not determine the moult state (i.e. missing feathers) were excluded from further analyses. All N_1_ and N_2_ feathers were sufficiently large for subsequent cort_f_ analysis. The age-related distribution of feather states across populations is shown in Fig. S1. After sampling, all birds were transferred to the experimental aviaries of the Station d'Ecologie Théorique et Expérimentale du CNRS in Moulis, France (42°57′ N, 01°05′ E).

### Challenge experiment

Animal welfare, maintenance and experimental procedures were followed according to the regulations and guidelines of the Direction Régionale de l'Environnement, de l'Aménagement et du Logement of France (permit number: 31-2014-09). All birds were kept in a modular outdoor aviary, of which each module (henceforth ‘cage’) measured 4 m×1 m with a height of 3 m and was equipped with adequate numbers of roosting boxes and bamboo plants for perching. Birds from each of the six sampling plots were randomly assigned to a diet mimicking either an urban diet (30% corn, 25% bread, 25% cake, 20% potato chips; henceforth ‘urban diet treatment’) or a rural one (49% corn, 24% wheat, 24% sunflower seed, 3% dried mealworm; ‘rural diet treatment’) which differed in nutrient composition [see Salleh Hudin et al., (2016) for details]. Each origin×diet treatment combination was replicated twice to account for cage effects. Hence sparrows caught in each of the six plots were assigned to one of four treatment groups, resulting in 24 experimental groups that comprised all possible combinations of bird origin and diet. The average group size per cage was 4.75±0.74 (range: 3–5) individuals, and age and sex distributions were kept as constant as possible across groups (Salleh Hudin et al., 2016). The length of the regrowing feathers was measured once every week and after 6 weeks, before release of the birds, fully regrown left and right fifth rectrices were collected. Growth time was defined as the number of weeks it took for the feather to complete regrowth after induced moult (range 3–6 weeks, accuracy 0.5 weeks). This value was derived from the correlation between week and feather length, for each feather separately. For example, if a feather showed linear growth until week 4 and then stopped growing, the growth time received a score of 4. Alternatively, when growth in feather length was linear until week 3 and slowed down between week 3 and 4, after which it did not increase any more, then growth time was given a score of 3.5. All individuals were released at their original capture site after completion of the experiment. Sample sizes of original and regrown feathers in relation to population of origin, age and sex are listed in Table S1 while the number of regrown feathers in relation to moult state of the original feather and treatment groups are shown in Fig. S2.

### Quantification of cort_f_

We applied an ultra-performance liquid chromatography coupled to tandem mass spectrometry (UPLC-MS/MS) method for corticosterone and its direct precursor (11-deoxycorticosterone), adjusted from Aerts et al., (2015), that was earlier shown to reduce the problem of cross-reactivity commonly encountered when using radio (RIA) or enzyme immunoassay (EIA) due to its high specificity.

Chromatographic analysis was performed on an Acquity UPLC-MS/MS Xevo TQS using an Acquity Ultra Performance LC BEH C_18_ (1.7 μm; 2.1 mm×100 mm) column (Waters, Milford, USA). Samples were evaporated to dryness with a Turbovap nitrogen evaporator (Biotage, Uppsala, Sweden). Grace Pure SPE C_18_-Max (500 mg, 6 ml) columns for solid-phase extraction (SPE) were obtained from Grace Davison Discovery Sciences (Lokeren, Belgium). High-performance liquid chromatography (HPLC)-gradient grade methanol (HiPerSolv Chromanorm) as extraction solvent was obtained from VWR International BVBA (Leuven, Belgium). Methanol absolute LC-MS as well as formic acid ULC-MS grade from Biosolve BV (Valkenswaard, The Netherlands) and ultrapure water of a Milli-Q from Millipore (Billerica, USA) were used as mobile phase solvents. Only products with a certificate of analysis were used. Corticosterone and 11-deoxycorticosterone were obtained from Sigma-Aldrich and corticosterone-d_8_ from CDN Isotopes (Pointe-Claire, Canada) was used as an internal standard.

A single feather, weighing on average 10 mg, was sampled and dirt (e.g. feces, mud, etc.) was manually removed by using tweezers. Meticulous cleaning of the feathers eliminated the possibility of dried blood from blood quills being analyzed (possibly biasing the analysis of N_1_ feathers; see higher), and no blood was visible on any feather sample. Next, each feather was flattened on a polystyrene board along a metal ruler, where needed, by pinning it to keep its position in order to measure its total length (cm). Subsequently, the weight of the feather was determined on an analytical balance XPE205 from Mettler-Toledo (Zaventem, Belgium). Using scissors, the feather was cut perpendicular to the rachis and just above the superior umbilicus to remove the calamus. Again, length and weight of the feather were determined. To obtain a homogenized sample, the feather was cut into fine pieces (<2 mm) using scissors. Between samples, scissors were rinsed with methanol, followed by ultrapure distilled water and dried with a paper tissue to avoid cross-contamination between samples. The amount of homogenized sample used for analysis was standardized at 10 mg. First, the sample was extracted with 8000 µl of methanol and 10 µl of a corticosterone-d_8_ solution of 0.5 µg l^−1^ was added as internal standard. When other amounts of sample were used, the results were corrected accordingly. Next, the sample was vortex-mixed for 30 s to homogenize and put on an overhead shaker for 60 min at 90 rpm. Subsequently, the sample was centrifuged for 10 min at 3452 ***g*** (=4000 rpm on a swing-out) at 7°C and the supernatant was transferred to a new 12 ml tube. The sample was evaporated to dryness under nitrogen at 60°C and resuspended in 5000 µl H_2_O/MeOH (80:20, v/v). After conditioning a C_18_ SPE column with 3 ml of MeOH followed by 3 ml of H_2_O, the sample was loaded. The column was washed with 4.5 ml H_2_O/MeOH (65:35; v/v) and retained compounds were eluted with 2.5 ml H_2_O/MeOH (20:80; v/v) into a 12 ml test tube and evaporated to dryness under a stream of nitrogen at 60°C. The sample was reconstituted in 50 µl H_2_O/MeOH (80:20; v/v) in a vial with insert and analyzed by means of UPLC-MS/MS. Since in future research matrix-matched calibration curves are not feasible, calibration curves were made in H_2_O/MeOH (80:20, v/v). Subsequently, the stock factor was 10000 and results were corrected for this. Results were reported as the value (ng/mg or µg/kg)±the expanded measurement uncertainty (U) (ng/mg or µg/kg) with a coverage factor (k) of 2 (95% confidence interval).

### Quantification of GBW and FA

Length, mass, GBW and FA of each original and regrown feather were measured by a single person (NSH) following the procedures outlined in (Salleh Hudin et al., 2016). Measurements were repeated resulting in two independent measurements per feather, which were highly repeatable (Salleh Hudin et al., 2016). Feather quality was estimated as the residuals from the regression between feather mass and feather length (r^2^=0.78, *N*=113 feathers) (De La Hera et al., 2010). Feather FA was analyzed through mixed-regression analysis with restricted maximum likelihood (REML) parameter estimation (van Dongen et al., 1999). In this model, the fixed intercept estimates overall trait size, the fixed slope estimates directional asymmetry (DA), and the random intercepts and slopes estimate the variation in individual trait value and individual FA, respectively (van Dongen et al., 1999). Variance due to measurement error (ME) was homogeneously distributed between sampling plots and treatment groups (likelihood-ratio tests: all *P*>0.05), hence a single error component was estimated. Variance in signed FA (δ^2^_FA_=0.4066) was more than tenfold larger than variance in ME (δ^2^_ME_=0.03085) and was highly significant (likelihood-ratio test: *P*<0.0001). FA measurements were not biased by DA (*F*_1,55_=0.16; *P*=0.695; denominator degrees of freedom computed using Satterthwaite's formula following Verbeke and Molenberghs, (1997). For hypothesis testing, unbiased FA values per individual were calculated as the variance components of the slopes of the individual regression lines in the mixed regression model.

### Statistical analyses

To test whether cort_f_ in original feathers differed between urban and rural populations (factor urbanization), Linear Mixed Models (LMM) were fitted. All models included moult state (old versus new feather; see Results for rationale), sex and age (juvenile versus adult) as fixed factors (both intrinsic factors may affect the activity of the HPA axis (Bonier, 2012; Bonier et al., 2007b; Dantzer et al., 2014), while population of origin was modelled as a random intercept. Since cort_f_ is passively deposited during feather formation and may therefore vary with feather length (Bortolotti, 2010; Jenni-Eiermann et al., 2015; Romero and Fairhurst, 2016), the latter was included as a covariate. Two-way interactions between fixed factors were also tested. To test whether cort_f_ in original feathers was related to GBW and FA, two separate LMMs were run.

We collected fully regrown feathers from 85 individuals, and sample sizes according to moulting stage of the homologous original feathers are shown in Fig. S2. To test whether the quality of regrown feathers varied with urbanization (urban or rural), diet treatment, sex and/or age, we ran LMMs including all two-way interactions between fixed factors. Moult state of the original feather and growth time of the regrown feather were included as fixed covariates, while cage (nested within population of origin) was included as a random factor. For 55 individuals for which we obtained cort_f_ from both their original and regrown feathers, we tested to what extent both values were correlated, and how this relationship varied with urbanization, diet treatment, moult state of the natural feather, age and/or sex. This analysis was performed on N_(1,2)_ and O original feathers only (see higher).

To test for differences in cort_f_ between sparrows of urban and rural origin in response to our experimental treatment, we applied a LMM including the level of urbanization in the population of origin (urban versus rural), the diet treatment, sex, age and all two-way interactions as fixed factors. Moult state of the original feather and length of the regrown feather were included as covariates (Jenni-Eiermann et al., 2015; Romero and Fairhurst, 2016), while cage (nested within population of origin) was included as random factor. Because cort_f_ values of original and regrown feathers were not significantly correlated (see Results), we did not include the former as a covariate, hence allowing a sample size of 85 individuals (results were highly comparable when including cort_f_ of original feathers as a covariate; results not shown). To estimate the level of correlation of cort_f_ with GBW and FA in regrown feathers, two separate LMMs were fitted, including urbanization, diet, sex, age and their two-factor interactions with GBW or FA as fixed factors.

Because between-year variation in environmental conditions may result in structural differences among feathers [and hence in cort_f_ deposition; (Patterson et al., 2015; Romero and Fairhurst, 2016)], we verified whether cort_f_ of original and regrown feathers was confounded with moult state by modelling adult feather properties (length, quality, GBW) in relation to moult state, urbanization and sex (LMM with population of origin included as a random factor). Females had shorter (*F*_1,29.9_=12.92, *P*=0.0012) and lower quality (*F*_1,31_=4.34, *P*=0.045) feathers than males, but none of the other relationships were significant (all *P*>0.29). Hence, environmental conditions during moult were apparently quite similar between years, even though moult status did vary with population of origin and age and was therefore included as covariate during hypothesis testing (comparable relationships were obtained if original and regrown feathers were modelled separately; results not shown).

During hypothesis testing, full models (i.e. those containing all explanatory variables outlined above) were reduced by excluding effects with the highest *P*-values until the AIC estimate of a reduced model did not decrease compared to the previous most parsimonious one. Interactions were always removed before main effects. Significant statistics and *P*-values mentioned in text and tables refer to the final minimal model, whereas statistics and *P*-values of non-significant terms were obtained by fitting each non-significant term separately into the minimal model. Model assumptions and residuals did not deviate from expectations in any of the models.

## Supplementary Material

Supplementary information
