# Correction: Do wild-caught urban house sparrows show desensitized stress responses to a novel stressor? (doi:10.1242/bio.031849)

**DOI:** 10.1242/bio.038513

**Published:** 2018-09-15

**Authors:** Noraine Salleh Hudin, Aimeric Teyssier, Johan Aerts, Graham D. Fairhurst, Diederik Strubbe, Joë l White, Liesbeth De Neve, Luc Lens

**Affiliations:** 1 Terrestrial Ecology Unit, Department of Biology, Ghent University, K.L. Ledeganckstraat 35, B-9000 Ghent, Belgium; 2 Department of Biological Sciences, Faculty of Science & Mathematics, Universiti Pendidikan Sultan Idris, 35900 Tanjong Malim, Perak, Malaysia; 3 Stress Physiology Research Group, Faculty of Pharmaceutical Sciences, Ghent University, Wetenschapspark 1, 8400 Ostend, Belgium; 4 Stress Physiology Research Group, Animal Sciences Unit, Flanders Research Institute for Agriculture, Fisheries and Food, Wetenschapspark 1, 8400 Ostend, Belgium; 5 Department of Biology, University of Saskatchewan, 112 Science Place, Saskatoon, Saskatchewan S7N 5E2 Canada; 6 Laboratoire Evolution & Diversité Biologique, UMR 5174 CNRS-Université Paul Sabatier-IRD-ENSFEA, 118 route de Narbonne, F-31062 Toulouse, France; 7 Center for Macroecology, Evolution and Climate, Natural History Museum of Denmark, University of Copenhagen, Copenhagen, Denmark

There were errors published in Biology Open (2018) 7, bio031849 (doi:10.1242/bio.031849)

D. Strubbe's second affiliation and funders were omitted from the original manuscript. This has now been amended in the online version.

The author apologises for any inconvenience.

