# Supplementary material for: Do wild-caught urban house sparrows show desensitized stress responses to a novel stressor?
Source: Biol Open. 2018 Apr 9;7(6):bio031849. doi: 10.1242/bio.031849 (PMC6031342; doi:10.1242/bio.031849)
Supplement: Supplementary information [file biolopen-7-031849-s1.pdf]

Supplementary Information

Table S1. Number of captured birds (Males *vs* Females; Adults *vs* Juveniles) in each population and number of regrown feathers that were collected after 6 weeks in captivity (number of captured birds/number of regrown feathers). A total of 113 birds were captured, of which 59.3% were males and 63.7% were adults. Regrown feathers could be collected from 87 birds.

**Origin**	**Urbanisation**	**Sex**		**Age**	
		Male	Female	Adult	Juvenile
**Caraman**	**Rural**	12/8	7/6	9/8	10/6
**Cologne**	**Rural**	9/9	8/7	7/7	10/9
**Montégut-Savès**	**Rural**	12/10	12/10	22/18	2/2
**Pau**	**Urban**	12/6	6/4	13/8	5/2
**Tarbes**	**Urban**	10/6	6/3	12/8	4/1
**Toulouse**	**Urban**	12/12	7/6	9/9	10/9
**TOTAL**		**67/51**	**46/36**	**72/58**	**41/29**

Table S2. Results from the LMM testing relationships between CORT$_f$ in original feathers and fluctuating asymmetry (FA) according to sex, age and urbanisation level. Effects that remained in the final model are shown in bold. Statistics and P-values of non-significant terms were obtained by fitting each non-significant term separately into the final model.

	DF	F	P
Main effects/covariates			
Feather Length	**1,48**	**70.49**	**<0.0001**
Moult state	1,47	0.54	0.465
**FA**	**1,48**	**1.92**	**0.173**
**Sex**	**1,48**	**4.45**	**0.040**
Age	1,46	0.16	0.690
Urbanisation	1,2.31	0.01	0.949
Interactions			
Urbanisation*FA	1,42.6	0.04	0.836
**Sex*FA**	**1,48**	**3.63**	**0.063**
Age*FA	1,44	0.02	0.899

Biology Open • Supplementary information

Table S3. Results from the LMM testing relationships between $CORT_f$ in original feathers and GBW, according to sex, age and urbanisation level. Effects that remained in the final model are shown in bold. Statistics and P-values of non-significant terms were obtained by fitting each non-significant term separately into the final model.

	DF	F	P
Main effects/covariates			
Feather Length	**1,58**	**65.51**	**<0.0001**
Moult state	1,57	0.06	0.81
GBW	1,54	1.27	0.26
Sex	1,57	0.00	0.97
Age	1,57	1.33	0.25
Urbanisation	1,57	0.17	0.68
Interactions			
Urbanisation*GBW	1,52	0.14	0.71
Sex*GBW	1,52	2.10	0.28
Age*GBW	1,52	0.13	0.72

Biology Open • Supplementary information

Table S4. Results from LMM explaining variation in the quality of regrown feathers. The final model is shown in bold. Statistics and P-values of non-significant effects were obtained by fitting each non-significant term separately into the final model. Estimates ± SE are given for Adults versus Juveniles, Females versus Males, Rural versus Urban sites and Rural versus Urban diet

Cage(origin) P=0.17

	Estimate	DF	F	P
Main effects/covariates				
Growth time	-1.096 ± 0.841	1,74.7	1.70	0.196
Moult state		4,74.5	2.00	0.103
Age	-0.182 ± 1.519	1,80.1	0.01	0.905
**Sex**	**-4.956 ± 1.394**	**1,70.1**	**12.64**	**0.0007**
**Urbanisation**	**-3.293 ± 1.660**	**1,15.1**	**3.93**	**0.066**
**Diet**	**-3.625 ± 1.636**	**1,14.9**	**4.91**	**0.043**
Interactions				
Sex*Age		1,79.1	0.06	0.813
Urbanisation*Diet		1,14	0.09	0.770
Urbanisation*Sex		1,68.9	1.40	0.241
Urbanisation*Age		1,78.9	0.30	0.588
Diet*Sex		1,70.5	1.38	0.244
Diet*Age		1,79.9	1.41	0.523

Biology Open • Supplementary information

Figure S1. The number of original tail feathers according to moult state, per population of origin. Bourgezes, Cox and Mont are rural areas, while Pau, Tarbes and Toulouse are urban areas. M1 - moulting feather not plucked, M2 – moulting feather plucked, N1 – New feather still growing, N2 – New feather fully grown, O – Old feather.

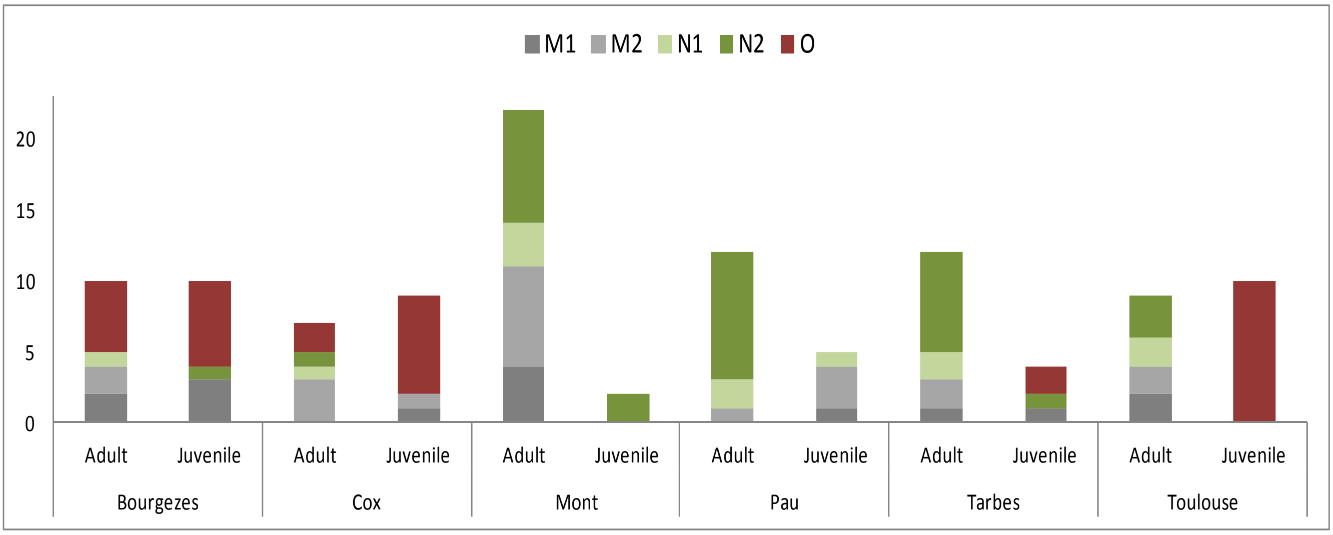

Biology Open • Supplementary information

Figure S2

Number of regrown feathers according to the moult status of the original homologous feather for each experimental treatment group. M1 - moulting feather not plucked, M2 – moulting feather plucked, N1 – New feather still growing, N2 – New feather fully grown, O – Old feather.

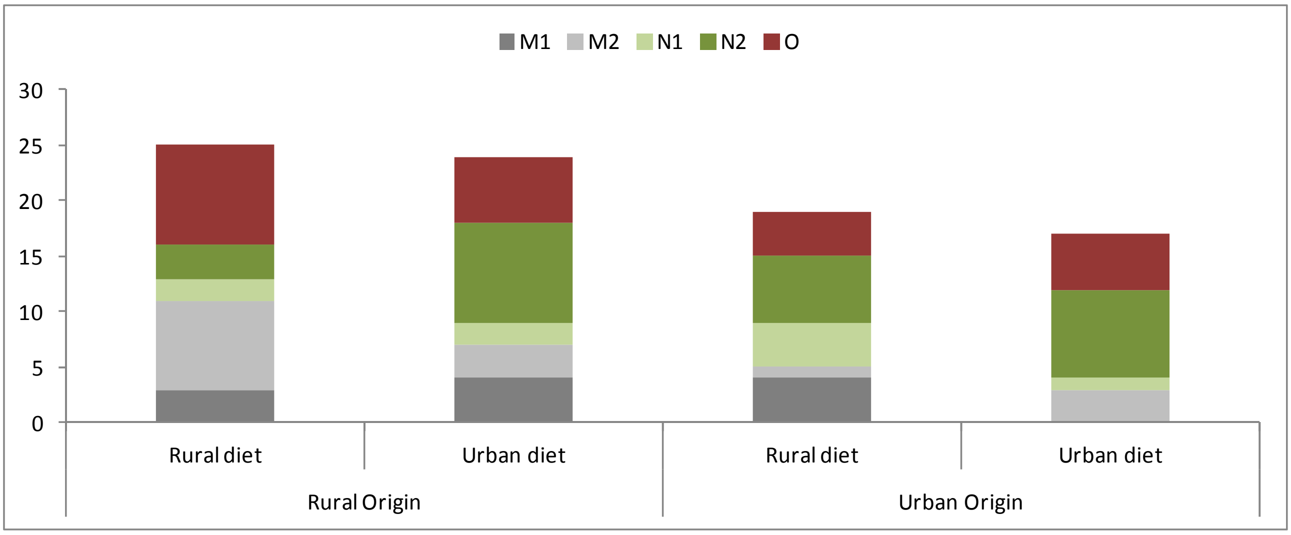

Biology Open • Supplementary information
